# Emerging links between cerebrovascular and neurodegenerative diseases—a special role for pericytes

**DOI:** 10.15252/embr.201948070

**Published:** 2019-10-16

**Authors:** Urban Lendahl, Per Nilsson, Christer Betsholtz

**Affiliations:** ^1^ Department of Cell and Molecular Biology Karolinska Institutet Stockholm Sweden; ^2^ Department of Neurobiology, Care Sciences and Society Division of Neurogeriatrics Center for Alzheimer Research Karolinska Institutet Solna Sweden; ^3^ Integrated Cardio Metabolic Centre (ICMC) Huddinge Sweden; ^4^ Department of Immunology, Genetics and Pathology Rudbeck Laboratory Uppsala University Uppsala Sweden; ^5^ Department of Medicine Karolinska Institutet Huddinge Sweden

**Keywords:** blood‐brain barrier, cerebrovascular disease, neurodegenerative disease, neurovascular unit, pericyte, Vascular Biology & Angiogenesis

## Abstract

Neurodegenerative and cerebrovascular diseases cause considerable human suffering, and therapy options for these two disease categories are limited or non‐existing. It is an emerging notion that neurodegenerative and cerebrovascular diseases are linked in several ways, and in this review, we discuss the current status regarding vascular dysregulation in neurodegenerative disease, and conversely, how cerebrovascular diseases are associated with central nervous system (CNS) degeneration and dysfunction. The emerging links between neurodegenerative and cerebrovascular diseases are reviewed with a particular focus on pericytes—important cells that ensheath the endothelium in the microvasculature and which are pivotal for blood–brain barrier function and cerebral blood flow. Finally, we address how novel molecular and cellular insights into pericytes and other vascular cell types may open new avenues for diagnosis and therapy development for these important diseases.

**Glossary**
20‐HETE: 20‐Hydroxyeicosatetraenoic acid
AD: Alzheimer's disease
ALK5: TGFβ type I receptor kinase
ALS: amyotrophic lateral sclerosis
APOE: apolipoprotein E
APP: amyloid precursor protein
Aqp4: aquaporin 4
atp: adenosine triphosphate
a‐v: arterio‐venous
avm: arteriovenous malformations
aβ: amyloid β peptide
BBB: blood–brain barrier
BDNF: brain‐derived neurotrophic factor
CAA: cerebral amyloid angiopathy
CADASIL: cerebral autosomal dominant arteriopathy with subcortical infarcts and leukoencephalopathy
CARASIL: cerebral autosomal recessive arteriopathy with subcortical infarcts and leukoencephalopathy
CBF: cerebral blood flow
Cldn: claudin
CNS: central nervous system
CSF: cerebrospinal fluid
CXCL12: C‐X‐C motif chemokine 12
CXCR4: C‐X‐C chemokine receptor type 4
ECM: extracellular matrix
EM: electron microscopy
FGF: fibroblast growth factor
GFAP: glial fibrillary acidic protein
Glut1: glucose transporter 1
HD: Huntington's disease
ISF: interstitial fluid
Lam: laminin
LRP1: low‐density lipoprotein receptor‐related protein 1
MCAM: melanoma cell adhesion molecule
MCI: mild cognitive impairment
NG2: neuron‐glial antigen 2
NVU: neurovascular unit
OPC: oligodendrocyte progenitor cell
PDGF: platelet‐derived growth factor
PDGFR: platelet‐derived growth factor receptor
PET: positron‐emission tomography
PVS: perivascular space
scRNA‐seq: single‐cell RNA sequencing
SMA: smooth muscle actin
SOD1: super oxide dismutase 1
SVD: small vessel disease
TBI: traumatic brain injury
TGF: transforming growth factor
tPA: tissue plasminogen activator
VSMC: vascular smooth muscle cell
WML: white matter lesions

## Introduction

### The brain and its vasculature

The human central nervous system (CNS) is composed of 100 billion neurons connected in intricate ways. In addition, there is an even larger number of supporting glial cells, i.e., astrocytes and oligodendrocytes, the latter forming myelin sheets around neurons and making up a large part of the white matter in the brain. Another important cell category is the microglia—macrophage‐like cells important for scavenging cellular debris and protein aggregates, but when activated they also become central players in brain inflammation. While neurons and glial cells traditionally have been classified into sub‐categories based on morphological and anatomical criteria or differences in neurotransmitter repertoire, a new and more fine‐grained view of neurons and glial cell sub‐types is now emerging as a result of recent transcriptomic analyses of the brain at the single‐cell level. Thus, more than 500 molecularly distinct classes of neurons and glial cells have been identified in the mouse brain 1, 2, and information about the cellular composition of specific brain regions is increasing rapidly 2, 3, 4, 5, 6, 7, 8. Insights into the developmental trajectories for the different cell lineages—proceeding from immature progenitors to specialized cells—are generated by computational analyses of single‐cell transcriptomes derived from different developmental stages 8, 9, 10. Most data thus far are derived from the mouse, but progress is made in understanding cell type diversity also in the human brain 10. With the available data from the normal unperturbed situation as a benchmark, gene expression changes related to diseases now start to be elucidated, for example, in Alzheimer's disease (AD) 11 and multiple sclerosis 12, where single‐nuclei RNA sequencing technology has been used to capture transcriptomes at single‐cell resolution. It is expected that several additional diseases, in particular those that can be faithfully recapitulated in mouse models, will be transcriptomically analyzed in a not too distant future.

**Figure 1:**
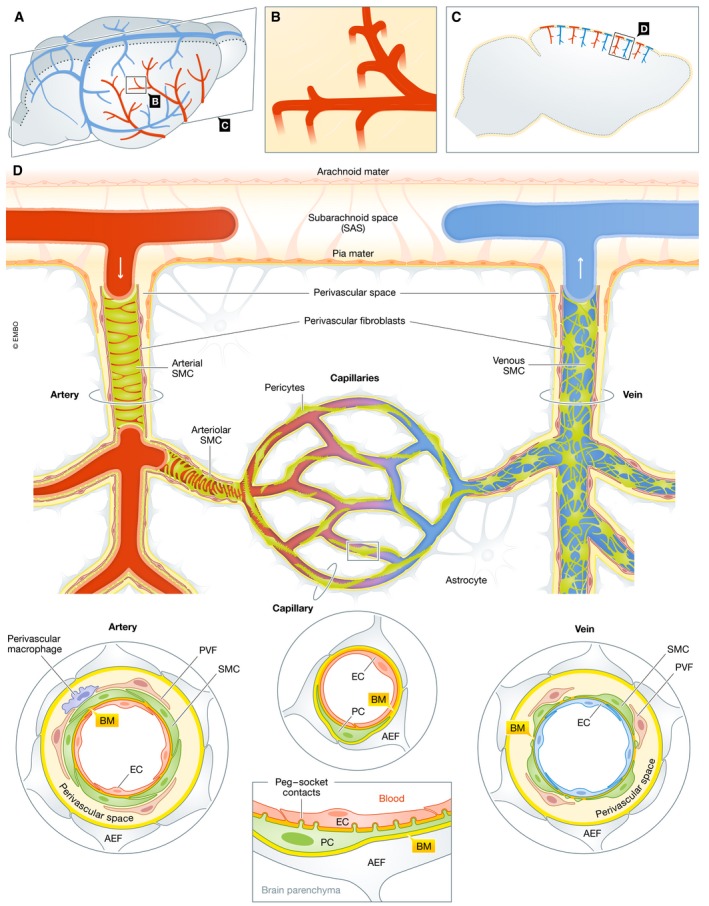
Overview of the brain vasculature (A) Schematic view of the dorsal surface of the mouse brain with feeding arteries (red) and draining veins (blue). (B) Branches from the superficial vessels, present in the subarachnoid space, penetrate perpendicularly into the brain parenchyma. (C) Schematic sagittal section of the brain at the level indicated in (A) with penetrating arterioles and veins depicted in the cerebral cortex. (D) High‐magnification schematic of a penetrating arteriole that branches into smaller arterioles and capillaries. The capillaries subsequently rejoin into radially organized venules, which drain into veins in the arachnoid. Schematic cross‐sections of the blood vessels at the arteriolar, capillary, and venous levels are indicated, depicting the various vascular and vessel‐associated cell types (as indicated in the figure), and the perivascular spaces that are important for CSF and interstitial fluid transport and follow the penetrating arterioles and veins. The longitudinal stretch of a capillary illustrates how a single pericyte connects several endothelial cells through its multiple peg‐socket contacts.

The brain constitutes ≈2% of the adult human body weight but receives 20% of the cardiac output and consumes 20% of our oxygen and glucose. However, it has almost negligible capacity for long‐term storage of energy and is therefore dependent on an elaborate vasculature that continuously fuels the brain with oxygen and nutrients. The vasculature of the brain has an intricate architecture: Feeding arteries follow the outer rim of the brain via the meninges and make branches that penetrate perpendicularly into the brain parenchyma (sometimes referred to as penetrating arterioles), where they further split into smaller arterioles and eventually into capillaries (Fig 1A–C). The capillaries subsequently join into venules that collect into radially oriented veins that further drain into the veins of the arachnoid (Fig 1D).

**Figure 2:**
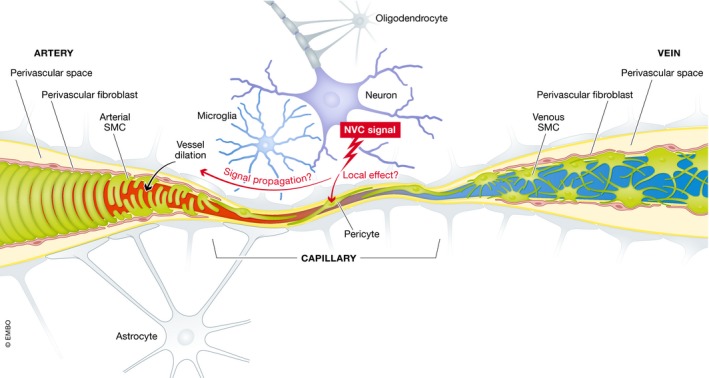
Pericytes and the neurovascular unit In the blood–brain barrier (BBB), endothelial cells are interlocked via tight junctions and adherens junctions, and extensively coated by mural cells. A blood vessel is depicted from the arterial side via a capillary to the venous side, with its coating of different vascular mural cells. The neurovascular unit (NVU) is indicated in the middle of the figure as a firing neuron sending signals (unknown in their chemical nature) to the local capillary branch, in which the pericyte may play a role as a receiver, in turn exerting local effects or upstream‐propagating signals that regulate smooth muscle cell contraction/relaxation at the terminal arteriole.

The blood vessels are built from two principal cell types: an inner lining of endothelial cells surrounded by an outer layer of mural cells. The mural cells have different phenotypes and are called by different names depending on vessel type: vascular smooth muscle cells (VSMC) cover arteries, arterioles, and veins, and pericytes cover capillaries and venules (Figs 1D and 2). The proportion of abluminal endothelial cell surface covered by mural cells varies among vessel types. The coverage is almost complete in arteries and larger veins, whereas it is incomplete in microvessels. The least pericyte coverage (≈10–20%) is observed in capillaries, where (in the brain) the pericytes are typically bipolar (sometimes tripolar at capillary branch points) and stretch two primary processes along the length axis of the capillary. From the primary processes, short sawtooth‐like secondary processes extend.

For the most part, the interface area between the pericytes and endothelial cells is physically separated by basement membrane. However, at specialized sites, often located at the end of secondary processes, the pericyte and endothelial cell membranes come into close apposition. These sites are referred to as peg‐socket contacts and adhesion plaques. In the peg‐socket contacts, pericytic cytoplasmic projections (“pegs”) protrude into endothelial membrane pockets (“sockets”), whereas the adhesion plaques are flat areas of contact. An important question related to both of these contact types is if one pericyte contacts one or more endothelial cells, thereby potentially integrating their functional behavior. The peg‐socket structures have thus far mostly been observed at the ultrastructural level in transmission or scanning electron microscopy analysis of animal tissues 13, 14, but they also seem to form under *in vitro* conditions during pericyte–endothelial cell co‐culture 15. Little is, however, known about the physiological role of the peg‐socket contacts, but it is an intriguing possibility that these structures harbor gap junctions and possibly also other moieties involved in contact‐dependent (“juxtacrine”) signaling 16 (Fig 1D). Although the pericyte:endothelial ratio in the brain is high compared to other organs, and sometimes claimed to be in the order of 1:1, our own unpublished observations suggest that the brain pericyte density is severalfold lower than that of endothelial cells. Therefore, from the fact that the contact between the capillary tube and pericytes in the brain is close to 100% (i.e., one would find a pericyte–endothelial interface area in every capillary cross‐section) follows that one pericyte contacts several endothelial cells (Fig 1B). Since pericytes are difficult to distinguish from other perivascular cell types using light microscopy based on singular protein or mRNA markers, the peg‐socket contacts may constitute a defining criterion for pericytes versus other perivascular cell types, including perivascular fibroblasts and macrophages. Progress in understanding pericytes and endothelial cells at the proteomic and transcriptomic levels may provide ideas for cytoskeletal and other proteins that reside in the peg‐socket contact and adhesion plaques.

### The blood–brain barrier

A particular feature of the brain vasculature is the blood–brain barrier (BBB), which serves to separate the blood from the extracellular fluids of the brain (interstitial and cerebrospinal fluid; ISF and CSF, respectively) and hinders pathogens and xenobiotic substances from entering the brain. The non‐fenestrated endothelial cells in the brain vasculature are interlocked via tight junctions and adherens junctions, forming a tight physical barrier. This contrasts with the endothelium in, for example, the liver, kidney, and endocrine organs, which is fenestrated and therefore freely permeable for solutes and small proteins. To allow for efficient, precise, and regulated transport of ions, sugars, amino acids, nucleic acids, lipids, and proteins, the BBB is equipped with a wide range of specific influx transporters, as well as receptors that engage in receptor‐mediated transcytosis. In addition, in order to bounce off unwanted and potentially neurotoxic substances present in the blood, including xenobiotics taken up from the gut, the BBB endothelium also expresses several efflux transporters that nonspecifically recognize and export small lipophilic molecules. This feature of the BBB is a major barrier against low molecular weight pharmaceuticals and poses a problem for efficient drug delivery to the brain.

Whereas the specific features of the BBB described above mostly can be attributed to the endothelial cells, surrounding peri‐endothelial cells also play important roles as inducers and regulators of the BBB properties of endothelial cells as well as by taking active part in the homeostatic functions of the BBB. The latter is suggested by the many transporter molecules that are specifically expressed in brain pericytes as compared to lung pericytes 17. Among the many different types of peri‐endothelial cells, including immune cells, fibroblasts, and—depending on the organ—epithelial cells, the pericytes deserve special attention because they appear to be a ubiquitous and obligatory component of the microvessel wall. As mentioned, the CNS harbors pericytes at higher density than in peripheral organs, for example, in skeletal muscle, where the ratio of pericytes to endothelial cells has been suggested to be a hundredfold lower than in the CNS (for review, see ref. 18). While the magnitude of the reported differences should be treated with caution since pericyte identification remains ambiguous, it appears that the pericyte density in the CNS is high enough to allow for contact with every (or at least the vast majority of) endothelial cell. The BBB integrity also depends on astrocytes, which almost completely encapsulate the vasculature with their endfeet 19 (Figs 1B and 2). Regulated transport across the endothelial layer in the BBB is largely driven by transcytosis in the endothelial cells, and as discussed below, the molecular underpinnings of controlled BBB permeability, and the role of pericytes, are intense areas of research.

Platelet‐derived growth factor signaling is critical for pericyte recruitment, differentiation, and homeostasis 18. Hence, evidence for the importance of pericytes for BBB integrity has mainly been provided from analyses of mouse models with hypomorphic PDGF signaling 20, 21. One such model, the *Pdgfb* ^ret/ret^ mouse, produces only a truncated PDGFB ligand (PDGFB‐ret) lacking a C‐terminal stretch of amino acid residues referred to as the retention motif. This motif mediates binding of the secreted PDGFB wild‐type molecule to proteoglycans on the cell surface or in the extracellular matrix 22. Truncation of the retention motif makes PDGFB‐ret unable to bind proteoglycans. While the PDGFB‐ret molecule retains its full capacity to activate the PDGF β‐receptor (PDGFR‐β), the increased diffusion of the PDGFB‐ret molecule away from its producer cells—the endothelium—presumably lowers its active concentration in the pericyte vicinity. In other models, the *Pdgfb* or *Pdgfrb* genes have been fully or partially ablated. In *Pdgfb* and Pdgfrb null mutants (heterozygous or homozygous) as well as in the *Pdgfb* ^ret/ret^ mouse, there is a reduction in pericyte numbers, coupled with BBB dysfunction and leakage as well as defects in endothelial transcytosis 20, 21, 23. More recent studies have demonstrated that the BBB leakage in the *Pdgfb* ^ret/ret^ mice differs in different brain regions 24. Pericyte loss has also been studied in another PDGF‐signaling impaired mouse model, the *Pdgfrb* ^F7/F7^ mice, which reduces signaling competence on the receptor side 25. In the *Pdgfrb* ^F7/F7^ mice, both pericytes and VSMC are affected, but BBB breakdown was observed in the *Pdgfrb* ^F7/F7^ mice at the stage when only pericytes were affected, indicating that the BBB impairment in these mice develops as a result of the loss of pericytes, rather than due to loss of VSMC 26.

Pericytes and endothelial cells are engaged in a complex cross‐talk, which is only partially understood. There are several ligand–receptor interactions operating between pericytes and endothelial cells, including PDGF‐PDGFR, Ang1‐Tie‐2, and TGFβ signaling 18. The Notch signaling pathway is also involved, notably by regulating PDGFR‐β expression in pericytes and VSMC 27. Accordingly, Notch signaling is pivotal for the integrity of pericytes and VSMC 27, 28, 29. Recent work has also implicated CD146 (MCAM) in pericyte recruitment, potentially as a co‐receptor for PDGFR‐β 30. While these and other studies 31 clearly show that the loss of pericytes impairs the formation of vascular barriers in the CNS, activated or perturbed pericytes may also do harm to the CNS vasculature 32. It has, for example, been demonstrated that the specific ablation of the TGFβ receptor ALK5 in pericytes causes brain hemorrhage in developing mouse embryos, but does so without depleting pericyte number 33. This suggests that modulation of a specific gene function in pericytes can result in a more severe phenotype than that observed in the complete absence of pericytes 34. Thus, pericyte dysfunction may have worse consequences for the BBB than pericyte dropout.

The complex role of pericytes at the BBB is underscored by studies of mice with reduced pericyte numbers, such as the above‐mentioned *Pdgfb* ^ret/ret^ mice, which nevertheless eventually develop a seemingly stable pericyte‐deficient brain vasculature with specific attenuation of the endothelial transcytosis barrier 20. The mechanism(s) involved in the transcytosis process activated by pericyte hypoplasia are unknown, however, and while insights into receptor‐mediated transcytosis mechanisms across brain endothelial cells were recently provided by a study revealing a system of intracellular tubules that control transport across the endothelial cell 35, it is unclear whether loss of pericytes affects this system. To what extent pericytes affect other aspects of the BBB, such as the endothelial junctions, is also unclear. Tight junction proteins such as claudins, and in particular the endothelial‐specific claudin 5 (Cldn5) 36, are important for BBB integrity and limit paracellular transport via interlocking endothelial cells by tight junctions (for review see ref. 37). Available data on whether pericyte loss (caused by the above‐mentioned *Pdgfb* and *Pdgfrb* mutants) leads to changes in Cldn5 expression are partly inconsistent 20, 38 and the topic therefore requires further study.

The brain needs to rapidly increase oxygen and nutrient supply to the brain areas that at a given point of time are most active. Thus, the local cerebral blood flow (CBF) needs to be constantly adjusted to increase blood flow to active brain regions. The neurovascular unit (NVU), which is a conglomerate of endothelial cells, pericytes, VSMC, astrocytes, and neurons, is an important regulator of CBF (Fig 2). The connection between neuronal activity and CBF, which allows for the adjustment of CBF in response to the extent of neuronal activity, is referred to as neurovascular coupling (a.k.a. functional hyperemia). The specific roles of the various vascular cell types in CBF regulation are only partially understood, and several cell types have been implicated in neurovascular coupling. There are studies advocating regulatory roles for pericytes 39, 40, 41 (for review see ref. 42), astrocytes (although via pericytes) 43 as well as VSMC 44. Erythrocytes themselves may also influence CBF via oxygen sensing of their own deformability 45.

These divergent views suggest that a deeper molecular understanding of pericytes and other cell types in the brain vasculature is warranted. The first transcriptomic maps of vascular cells were generated by RNA sequencing at the cell population level 46, 47, 48. More recently, single‐cell RNA‐sequencing (scRNA‐seq) was used to provide transcriptional profiles for pericytes and all other brain vascular cell types, as well as for astrocytes and microglia 17. The single‐cell transcriptomes of brain endothelial cells reveal a graded transcriptional zonation from the arterial to the venous side, which matches with the gradual change in vessel diameter and presumed changes in biophysical parameters such as pressure and sheer, and chemical composition of the blood such as level of oxygenation, that takes place along the arterio‐venous (A‐V) axis. In contrast to the gradual transcription changes in endothelial cells, mural cells showed a more distinct zonation pattern along the A‐V axis. Here, pericytes constitute a quite distinct cell population, well‐separated from arterial and arteriolar VSMC transcriptional profiles, but with a gradual phenotypic conversion into venous VSMC. An intriguing finding was the abrupt transition from the arteriolar SMC phenotype to a capillary pericyte phenotype from one cell to the next at the border between a terminal arteriole and the following capillary 17. Previous reports have shown that pericytes in the mouse brain exhibit distinct morphologies depending on their location: a pericyte located on the capillaries is, for example, morphologically distinct from a pericyte on the postcapillary venules (for reviews see refs. 18, 49; Fig 2). It was therefore an unexpected finding that the brain pericytes in the scRNA‐seq study constituted a molecularly homogenous population of cells without signs of molecular subtypes 17. The apparent discrepancy between transcriptional homogeneity and morphological heterogeneity of pericytes is not understood. The pericytes in the scRNA‐seq study were captured from different brain regions via a dual reporter system (fluorescent reporters driven by PDGFRβ and NG2 regulatory elements) 17, and although dual‐labeled pericytes can be identified in all main areas of the brain, it remains possible that some subpopulations were not efficiently captured by this strategy. Alternatively, some pericyte populations may fare less well in the sorting and enrichment procedures, leading to underrepresentation in the transcriptome data set. With the advent of new pericyte markers 17, the question of heterogeneity can now be systematically addressed. Furthermore the discovery of novel ways to specifically stain pericytes, for example by Neurotrace 500/52 50, may allow for complementary scRNA‐seq explorations of brain pericyte transcriptomes. The pericyte scRNA‐seq analysis revealed discrepancies between certain mRNA species and the reported expression levels of the cognate protein. Notably, expression of α‐smooth muscle cell actin (*α‐SMA*) (*Acta2*) mRNA was very low in brain pericytes, which adds to the active discussion on the distribution and role of α‐SMA in pericytes. While some reports fail to detect α‐SMA immunoreactivity in the pericytes of a healthy mouse CNS 17, 44, other reports have identified α‐SMA immunoreactivity 51, 52, 53 as well as reporter gene activity driven from the α‐SMA promoter 44 in brain pericytes. α‐SMA levels may differ depending on pericyte location: pericytes closer to the arteriolar side may express higher α‐SMA levels, while pericytes at the center of the capillary bed express less α‐SMA 51. It has also been proposed that α‐SMA is rapidly degraded during sample preparation, which may be a reason why it is not detected by immunohistochemistry in all situations 54. The reason for the very low level of *α‐SMA* mRNA expression from the transcriptomic analysis versus higher protein levels observed by immunohistochemistry remains to be explored, and such information will be important for the understanding of the putative contractile properties of pericytes.

### Paravascular flow

**Figure 3:**
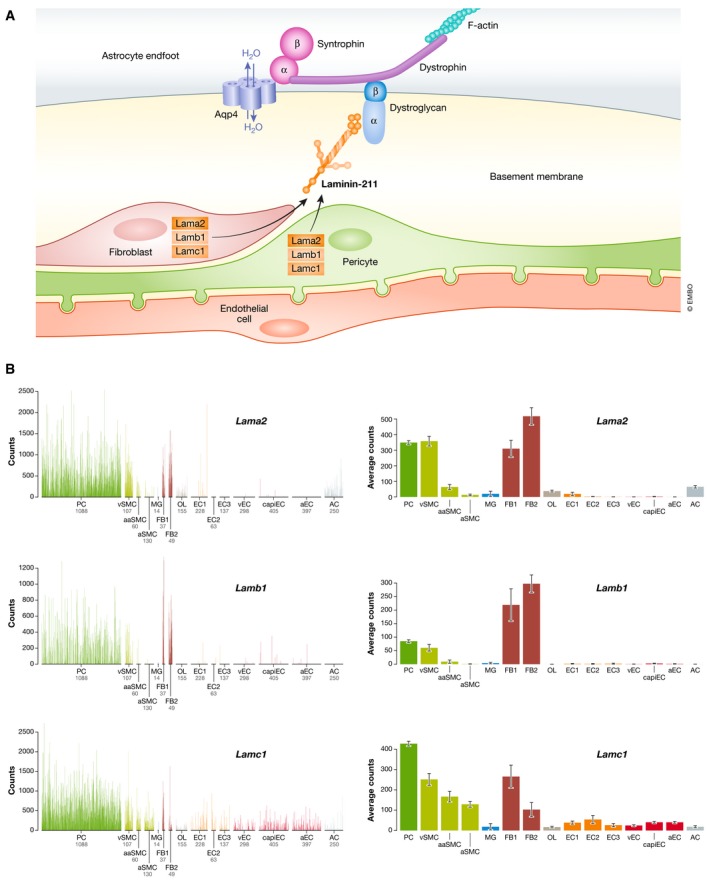
Pericytes and the glymphatic system (A) A schematic model for the interaction between pericytes and the glymphatic system. The figure shows that pericytes (PC) and perivascular fibroblasts (FB) express *Lama2*,* Lamb1,* and *Lamc1*, which encode the subunits of laminin 211. Laminin 211 interacts with dystrophin in astrocytes. This leads to the concentration of aquaporin 4 (Aqp4) in the astrocytic endfeet, which is important for the fluid transport and the function of the glymphatic system. (B) The mRNA expression profiles for *Lama2*,* Lamb1,* and *Lamc1* (from http://betsholtzlab.org/VascularSingleCells/database.html 17) are depicted.

In addition to the vascular system proper, there is a second major important fluid‐transporting system in the brain—paravascular flow—regulating the flow of CSF and ISF in the brain parenchyma (Fig 1). CSF is generated in the choroid plexus, transported via the subarachnoid space in the meninges, and drained via specialized connections to veins and lymphatic vessels present in the dura mater 55. The subarachnoid space is continuous with the brain's perivascular spaces (PVS), thin cavities that surround and follow the penetrating (parenchymal) arteries and veins 56, 57 (Fig 1D). The PVS are important for water, solute, and waste product exchange between the CSF and ISF. How the paravascular flow is regulated is only partially understood, but cerebral vascular basement membranes are important as conduits for fluid transport into and out of the brain 58. Furthermore, a recent report shows that experimental damage to the meningeal lymphatic system leads to accumulation of protein aggregates in the brain parenchyma including both AD‐associated amyloid β peptide (Aβ) 55 and tau 59, indicating that ISF and CBF drain to the meningeal lymphatics. An alternative system for paravascular flow is the glymphatic system, which has been proposed to be important as a removal system for products such as lactate, Aβ, and tau 60. In the glymphatic system, astrocytes are important for ion buffering and fluid exchange between the CSF and ISF, and it has been proposed that glymphatic clearance is higher in the sleep state 61. From this follows that sleep might be required for “waste” removal from the brain. The exchange through the glymphatic system has been suggested to be dependent on the water channel aquaporin‐4 (Aqp4) present in the astrocytic endfeet (Fig 3A). The role of Aqp4 in glymphatic function is, however, still a matter of discussion. The development of a functional glymphatic system in mice was shown to be correlated with the expression of Aqp4 at the astrocyte endfoot–vascular interface 62, and using several lines of Aqp4‐deficient mice, glymphatic tracer transport was shown to depend on intact Aqp4 function 63. It has, however, also been argued that Aqp4 may not be solely responsible for fluid transport, but that diffusion is in fact an important regulator of paravascular flow. In support of this notion, CSF tracer uptake and interstitial flow rate were unaffected by cardiac arrest or ablation of the *Aqp4* gene 64. Diffusion‐driven transport of injected tracers was also noted by several other studies 65, 66, 67, 68 (for review see 69). Tracer flow does not connect to perivenous drainage, and studies of the routing of tracers show that tracers injected into the CSF enter along pial‐glial membranes but exit the brain along VSMC basement membranes, going against the direction of blood flow 70. In addition, levels of ISF and CSF tau relate to neuronal activity and the sleep–wake cycle rather than to paravascular clearance 71. It has also been shown that the majority of Aβ removal (85%) occurs via the BBB and only a minority via ISF bulk flow in mice 72, 73, and similar findings are reported from humans 74. From these partially conflicting views on how paravascular flow is organized, and the role of the glymphatic system, it is clear that more research is required to understand in detail how fluid transport in the brain is regulated in the healthy and diseased states.

Do pericytes play a role in the glymphatic system? A recent analysis of pericyte‐deficient *Pdgfb* ^ret/ret^ mice shows a defective development and persistence of glymphatic function, suggesting that PDGFB signaling plays an important role in the development of the glymphatic system 62. It is likely that this role is mediated by pericytes, since the local absence of pericytes in *Pdgfb* ^ret/ret^ mice correlates with relocation of Aqp4 from astrocyte endfeet to the astrocyte cell soma at these sites 20. However, it should be kept in mind that the development of the vasculature in the *Pdgfb* ^ret/ret^ mice is more generally altered, including capillary dilation and impaired BBB function 20. Thus, pericytes may not only locally influence the endothelial cells, but may also interact with and regulate functions in neighboring astrocytes. Pericytes highly express laminin‐α2 (Lama2), laminin‐β1 (Lamb1), and laminin‐γ1 chains (Lamc1) 17 (Fig 3B), which, as part of laminin 211 deposited in the vascular basement membrane, is known to bind the dystrophin–glycoprotein complex in astrocytes, which in turn acts as a molecular bridge to Aqp4, promoting its concentration at the astrocyte endfeet 75 (Fig 3A). In keeping with a role for pericytes in the polarization of Aqp4 to astrocyte endfeet, knockout of the *Lama2* gene in mice causes BBB abnormalities and loss of Aqp4 polarization to astrocyte endfeet 76, similar to the situation reported in pericyte‐deficient mice. Thus, several studies support a scenario in which pericytes influence the development of the glymphatic system through deposition of laminin 211 in the vascular basement membrane, which via dystroglycan and dystrophin in astrocytes promotes polarization of Aqp4 to its endfeet.

In addition to molecularly defining the different mural cell subtypes, the scRNA‐seq analysis of the brain vasculature also identified a novel fibroblast‐like cell type—the perivascular fibroblast—with a localization close to or within the PVS 17 (Fig 1D). These cells differ from mural cells by their expression of PDGF receptor alpha and numerous extracellular matrix proteins in common with lung fibroblasts, including the fibrillar collagens 1,3, and 5, lumican, decorin, and others. They also deposit specific extracellular matrix (ECM) proteins, including laminin‐α1 (Lama1), which helped pinpointing the exact localization of these cells, namely surrounding arteries, arterioles, venules, and veins, but notably not around the capillaries. The localization of the perivascular fibroblasts along the PVS may be of relevance for paravascular flow, as certain aspects of the flow occur along vascular basement membranes (see above). An intriguing notion is that the perivascular fibroblasts reside between the two basement membranes—the astrocyte‐derived pial basement membrane and the basement membrane embedding the arteriolar and arterial VSMC— implicated in inward (arachnoid‐to‐brain) and outward (brain‐to‐arachnoid) tracer transport, respectively, indicating a possible role for the perivascular fibroblasts in the separation and compartmentalization of these tightly apposed transport routes 70. Notably, the perivascular fibroblasts abundantly express Lama2, Lamb1, and Lamc1, thereby perhaps also playing a role in astrocyte endfoot polarization around larger vessels, similar to the role of pericytes in the capillaries. The Lama1‐positive adventitial coat was independently observed in a recent study, although with a more restricted distribution on the venous side 77, and this difference in distribution should also be addressed in future studies. The localization of the perivascular brain fibroblasts along the PVS is also interesting in the light of cerebral small vessel disease (SVD) (see below). Expression of transporter proteins in the perivascular brain fibroblasts 17 may play a role in fluid composition and dynamics in the CSF and ISF and in the apposed opposite paravascular transport routes discussed above. Cells with a transcriptome profile closely resembling the perivascular brain fibroblast have been observed in other transcriptomic analyses of brain cells 2, 6 and additional, but not yet fully characterized, fibroblast subtypes have been identified 2, 17, indicating a broader fibroblast landscape within and around the CNS. It will be of interest to establish whether cells with characteristics similar to the perivascular brain fibroblast exist in the human brain. Earlier electron microscopy (EM) studies of human meningeal blood vessels 78 reveal a cell type with similar location and morphological characteristics, suggesting this to be the case, but a more detailed molecular analysis has not yet been performed.

## Neurodegenerative diseases and the brain vasculature—an emerging relationship

There is an emerging view that neurodegenerative diseases are characterized not only by decaying neuronal cells but also by vascular aberrations, and conversely, that diseases with an origin in the brain vasculature affect the surrounding CNS. To establish whether the CNS and vascular phenotypes represent incidental co‐occurrences or whether there are casual relationships between a dysfunctional CNS and problems in the brain vasculature is an important topic to address, with high relevance for understanding the pathobiology of the disease and to develop novel therapeutic strategies.

### Neurodegenerative diseases affecting the vasculature

Neurodegenerative diseases cause considerable human suffering and societal costs, and their incidence is increasing because of the aging population. In neurodegenerative diseases, specific types of neurons are lost, and insights into the underlying molecular pathology are rapidly accumulating. AD is characterized by loss of neurons and synapses in the cerebral cortex and hippocampus, and accumulation of Aβ peptide (from aberrant processing of the amyloid‐precursor protein (APP)) and tau in neurofibrillary tangles are hallmarks for AD. In a minority of patients, mutations are found in *presenilin* and *APP* genes, but most cases are sporadic, with a specific *APOE* allele (*APOE4*) as risk factor 79. Although the neuronal loss is a major factor for the cognitive decline, it is becoming increasingly appreciated that AD also has a vascular component 80, 81, 82, 83. At the ultrastructural level, pericytes are disorganized in AD and exhibit mitochondrial abnormalities, pinocytotic vesicles, and accumulation of osmophilic material 84, 85, which may impair BBB function. Furthermore, AD patients, as well as AD mouse models, show pericyte loss, which correlates with the extent of BBB degradation 86, 87. BBB breakdown is accelerated in AD 88 and is also observed in mild cognitive impairment (MCI) 89, 90. Overall, at least 40% of AD patients have pronounced vascular changes, and many of these patients will be diagnosed with a mixed type of dementia 91, 92, 93. Wolters *et al* 94 have unraveled links between cerebral perfusion and the risk of dementia. In line with this, CBF is reduced in AD 95, 96, 97, which may be attributed to morphological changes in capillaries 98, 99. A decline in the number of pericytes may be observed not only in the diseased condition but may be a part of the normal aging process, as BBB breakdown and pericyte loss increase in the aging hippocampus 89. The pericyte decline is linked to an increase in the levels of soluble PDGFRβ in CSF, and elevated soluble PDGFRβ levels in CSF are an early independent biomarker of cognitive dysfunction, irrespective of Aβ and tau status in the CSF 90. A consequence of the deterioration of pericytes and loss of BBB integrity is an increased influx of immune cells into the brain, which in turn drives inflammation, an important disease‐promoting factor in neurodegenerative as well as cerebrovascular disease (for review see 100). In addition to a role in BBB integrity, pericytes may be important for clearing aggregated Aβ, as pericyte‐loss leads to elevated Aβ levels, presumably by reducing clearance of Aβ from the ISF 87. Aβ may, however, also be toxic to pericytes 101, notably with an increased vulnerability if the pericytes are of the *ApoE4* genotype. The ApoE expression levels are allele‐specific with the lowest being for ApoE4 102. Pericytes can take up Aβ via LRP1 receptors, and the LRP1‐mediated uptake was inhibited by blocking pericyte ApoE production. The blockage could be rescued by supplementing with exogenous ApoE3 but not ApoE4 103, which is interesting given the role of the *APOE4* allele as risk factor in AD. *App* transgenic mice exhibiting high levels of Aβ40 show defects in vasodilatory response revealing that Aβ40 has toxic effects on the vascular system 104. The increased Aβ levels in AD lead to Aβ deposits not only in the brain parenchyma but also at the vessel wall in cerebral amyloid angiopathy (CAA). CAA, which is classified as an SVD (see also below), was found in more than 80% of all AD cases. However, Aβ deposits in the vascular system are also observed during normal aging process but for unknown reasons do not induce pathology (reviewed in ref. 105). CAA occurs in the neocortical and leptomeningeal arteries as well as in capillaries 106 and shows an increased Aβ40/42 ratio, which is different from that in Aβ deposits in the parenchyma, indicating a vascular‐specific mode of aggregation. Importantly, the Dutch, Iowa, Arctic, Flemish, and Italian familial autosomal dominant mutations in the *APP* gene, which change the Aβ sequence at amino acid 22, induce a strong CAA characterized by pronounced Aβ40 deposits in the vessel walls (reviewed in ref. 107). Interestingly, the CAA pathology has recently been recapitulated in *App* knock‐in mice harboring the Dutch mutation 108, indicating that further insights may be provided from animal experiments. Similar to the CAA and AD situation in humans, these mice exhibited microhemorrhages and reduced CBF. Transverse aortic constriction of the left common carotid artery to reduce blood flow to the brain of the knock‐in mice significantly enhanced CAA, implicating a tight connection between CAA and CBF.

In *APOE4* carriers, CAA is additionally observed in capillaries and frequently in arterioles, and this aggravated form of the disease is referred to as CAA type I 105. As discussed above, this may be linked to the *APOE4* allele‐specific vulnerability of the pericytes to Aβ and changes in the transport of Aβ over the BBB. However, astrocytes are also major contributors to ApoE production, and in the presence of CAA, they exhibit pathological changes such as swelling of their endfeet. In severe cases of CAA, an altered ionic and water balance is observed due to changes in potassium and water channels and eventually astrocyte loss will occur 109, which may be aggravated by the extensive neuroinflammation in AD brains. Eventually, CAA leads to degeneration of all vascular cells, including pericytes, which can cause microhemorrhages and infarction thus further aggravating the disease state. There are studies indicating that the vascular phenotype is an early event in the disease process, and that microvascular changes in fact precede cognitive impairment in a mouse model for AD 110. An early vascular impact is corroborated by data from a recent study identifying early cerebrovascular abnormalities in AD patients 97.

Amyotrophic lateral sclerosis (ALS) is a neurodegenerative disease characterized by loss of motor neurons, and mutations in more than 30 genes, including superoxide dismutase 1 (SOD1), have been identified in the disease 111. Breakdown of the blood–CNS barrier in the spinal cord, as evidenced by erythrocyte extravasation, is observed in ALS patients, and the number of pericytes can be reduced by almost 50% 112. It is not yet established whether similar changes occur in the motor cortex of the brain. Vascular problems, in the form of cerebral arteriovenous malformations (AVMs), have been associated with ALS development in a few cases 113, although it remains a possibility that initial treatment of AVMs might induce ALS‐like symptoms 114. Microvascular structures in the retina have been reported to be altered in ALS 115. ALS patients have elevated PDGFC expression, and activation of the PDGF‐CC signaling pathway via PDGFR‐α leads to an earlier onset of neurodegeneration in ALS mouse models 116, suggesting that modulation of this signaling pathway may be a potential therapeutic strategy for ALS.

Huntington's disease (HD) is a neurodegenerative disease caused by mutations in the Huntingtin gene, leading to CAG repeat/poly‐glutamine expansions 117. In HD, the expanded huntingtin proteins form protein aggregates in neurons, and basal ganglia, caudate nucleus, and putamen are among the first brain areas to be affected. Spiny neurons are also lost in the striatum. Recently, it has been noted that brain pericytes become activated early in the HD disease process 118, and HD is associated with impaired BBB function and decreased *PDGF‐β* mRNA expression in pericytes 119. Furthermore, blood–spinal cord barrier leakage is observed in HD patients 120.

In addition to the neurodegenerative diseases, vascular problems are also observed following traumatic brain injury (TBI) and in neuropsychiatric disorders (for review see 121). BBB disruptions occur in TBI 122, 123, which may contribute to the more long‐term neurological problems in TBI. This may be linked to pericyte dysfunction as pericyte migration away from the vascular wall was noted after experimental TBI in rats 124. PDGFRβ‐expressing cells, suggested to be pericytes, also increase in numbers in the trauma zone following experimental TBI in mice 125. In addition, deletion of *Aqp4* aggravates the TBI‐induced tau pathology in TBI mice, indicating a role for the glymphatic system in the clearance of tau 126. BBB dysfunction was likewise observed in a boxer suffering from chronic traumatic encephalopathy 127. Another emerging research area at the neuronal–vascular interface is schizophrenia, a mental disorder with complex underlying genetics. The cause of schizophrenia is not well‐understood but may involve disturbed dopamine and NMDA receptor signaling. In addition to the neurological problems, an aberrant microvasculature as well as abnormal CBF is observed in schizophrenia 128, 129, 130. Elevated levels of albumin in the CSF in schizophrenic patients indicate leakage across the BBB 131. In keeping with a leaky BBB, the level of Cldn5, one of the proteins in the endothelial cell tight junctions in the BBB (see below), was reduced in schizophrenic patients 132.

### Cerebrovascular diseases affecting the CNS

In the previous section, we discussed vascular complications in neurodegenerative diseases and specific roles for pericytes in this group of disorders, and we will now review evidence for the converse situation, i.e., vascular diseases giving rise to CNS dysfunction. There is obvious widespread damage to the CNS resulting from a massive stroke, but more confined vascular problems also affect the CNS. An important subgroup of cerebrovascular diseases is the cerebral small vessel diseases (SVDs), which account for approximately 20% of all ischemic strokes. SVDs are characterized by changes in the small vessels in the brain accompanied by enlarged PVS 133, 134. Importantly, damage to cerebral white and deep gray matter is also observed in SVD patients, indicating that the oligodendrocyte lineage and myelin production may be affected. Cerebral autosomal dominant arteriopathy with subcortical infarcts and leukoencephalopathy (CADASIL) is the most frequent monogenic form of SVD and is caused by mutations in the *NOTCH3* gene 135, leading to NOTCH3 protein accumulation, white matter lesions (WML), and subcortical infarcts. In CADASIL, the vascular changes appear to precede WML, suggesting a primary origin in the vasculature, which is in line with the fact that NOTCH3 is predominantly expressed in VSMC and pericytes. Interestingly, another genetic SVD, CARASIL, which is predominantly caused by loss‐of‐function *HTRA1* mutations 136, may be functionally linked to CADASIL, as trapping of the HTRA1 protein, a secreted serine protease, has been observed in NOTCH3‐containing CADASIL protein aggregates 137, arguing that the protein aggregates in CADASIL may serve as a molecular sink for HTRA1. Interestingly, the *Htra1* gene is predominantly expressed by astrocytes in mice and may exert its normal function in the PVS implicated in glymphatic function 17. CAA is classified as an SVD 133, but has, as discussed above, close bearings to AD (for review see 138), which further underscores the similarities between neurodegenerative and vascular diseases. How the vascular damage in any of these diseases leads to WML is not well‐understood, but an intriguing idea is that there is a direct relationship between cells in the vasculature and cells in the oligodendrocyte lineage, which produce myelination in the white matter 134. Expression of the chemokine ligand CXCL12 from the vasculature has been proposed to activate CXCR4 receptor signaling in oligodendrocyte progenitors 139, which may provide such a vasculature–white matter link. The notion of a vascular white matter cross‐talk is supported by several additional reports. Notably, BDNF and FGF produced by endothelial cells promote survival and proliferation of oligodendrocyte progenitors 140, for review see ref. 141. Similarly, OPC migrate along the vasculature during CNS development in response to endothelial cell‐derived CXCL12, which acts on CXCR4 on the oligodendrocyte progenitors 142. Direct interactions between pericytes and oligodendrocyte progenitors 143, as well as a role for pericytes in oligodendrocyte differentiation 144, have also been proposed, and a putative perivascular source of oligodendrocyte progenitors has been identified that can be activated following oligodendrocyte progenitor ablation 145. To what extent these mechanisms are altered in SVD will be an interesting avenue for future research.

Which additional molecular mechanisms may get derailed in the brain vasculature in SVD? PDGF signaling is, as discussed above, a key signaling mechanism in pericytes but there are also other signaling mechanisms important for the BBB and pericytes, including angiopoietin‐1/Tie‐2 and TGFβ signaling 18. A link between PDGF and TGFβ signaling has recently been presented, where TGFβ expression after experimental stroke was reduced in mice with compromised PDGF signaling 146. Furthermore, as discussed above, ALK5 signaling in pericytes upregulates TIMP3 expression, leading to enhanced metalloproteinase activity 33. Enhanced activation of matrix metalloproteinases at pericyte somata has also been reported in response to experimental occlusion of cortical capillaries 147. An interesting risk factor for SVD is mutations in the transcription factor *FOXF2*, which is expressed in pericytes and endothelial cells 148. In line with this, deletion of *Foxf2* in the mouse affects pericyte differentiation and BBB integrity 149.

Pericytes are, in addition to controlling BBB integrity, likely involved in the aftermath of an ischemic stroke, in which the blood flow to the brain is suddenly disrupted by a post‐stroke CBF reduction in the microvessels. This capillary constriction exacerbates the ischemia, and damage to the brain and pericytes have been implicated in the post‐stroke CBF regulation and capillary constriction. It was proposed that pericytes constrict capillaries after reduction in ATP levels (chemical ischemia) 40 and die after capillary constriction, presumably in a constricted state 40, 150. Mechanistically, ischemia‐induced pericyte constriction can be relieved by suppressing oxidative‐nitrative stress 52. Furthermore, in the heart, capillary blockage following coronary artery occlusion colocalized with pericytes, and treatment with adenosine, which relaxes pericytes, increased perfusion, corroborating a role for pericytes in the post‐stroke blood flow reduction 151. Considering some current controversies and, seemingly, contradictions in the published data, it is important to remember that the mentioned studies define mural cells as pericytes rather than VSMC based on the diameter of the vessels they surround. This contrasts with our recently published molecular definition of pericytes and discrimination between pericytes and VSMC that are based on single‐cell RNA‐sequencing 17. Importantly, these different definitions of where the exact anatomical “border” should be drawn between an arteriole and a capillary is directly related to the controversy about whether brain pericytes are α‐SMA‐positive or not 51. By applying the same criteria for mural cell classification as we did in our single‐cell study, Hill *et al* 44 concluded that arteriolar SMC—not pericytes—are responsible for the microvessel constriction following brain ischemia. While the controversies about mural cell contractility after stroke might boil down to different nomenclature, rather than conflicting data, further research is required to precisely define the role of the various mural cell types in post‐stroke capillary constriction.

In addition, pericytes have been proposed to shift their cellular phenotype toward microglia 152 or cause an accumulation of ECM following stroke 150. Pericyte loss has also been observed in premature infants 153, and it will be interesting to learn how this relates to the effects on brain function that in some cases are observed later in life after premature births. Pericytes have been implicated in scar formation in the brain following cerebral ischemia. Proliferation of PDGFRβ and CD105‐positive stromal cells, which may be derived from a sub‐population of pericytes, was observed following experimental ischemia in mice, and these cells would deposit ECM leading to post‐ischemic scarring 150. The stromal cells have a similar marker profile as cells involved in scar formation in the spinal cord, which also are proposed to be pericyte‐derived 154, 155. While these cells share several markers with pericytes, it has, however, been proposed that the cells involved in the scar process instead are perivascular fibroblasts 156. The molecular characterization of pericytes and perivascular brain fibroblasts in the mouse brain may support the latter notion 17, but more research is required to establish the precise nature of the cells involved in scar formation in the brain and spinal cord.

Enlarged PVS are a hallmark of and diagnostic criterion for SVD 157, but the reason why PVS are enlarged in SVD and a number of other brain diseases is poorly understood. It may be that the enlargement causes impaired paravascular flow, leading to reduced clearance, or conversely that accumulation of Aβ and other aggregates leads to the observed expansion of the PVS (for review see ref. 57). Alternatively, PVS enlargement may be caused by inflammation, for example, in multiple sclerosis 158, and in line with this idea, inflammatory cells are indeed observed in the PVS 57. Recent data reveal a genetic component for PVS enlargement, at least in certain regions of the brain (the basal ganglia) 159, and this genetic link could be a starting point for a search for genes implicated in PVS organization and function. The recent characterization of perivascular brain fibroblasts that are located along the PVS 17 (see above) may contribute to a better understanding of the cellular architecture of the PVS. The function of these cells is not yet well‐understood, but it is notable that they express transporters as well as specific ECM components, indicating that they may play a role in regulating paravascular flow. While these cells thus far have only been analyzed in detail in the mouse, it is, as discussed above, reasonable to assume that they also exist in the human brain, based on histological analysis of human brain blood vessels, which identifies cells with a similar localization around blood vessels 78. The identification of genes uniquely expressed in the perivascular brain fibroblasts may stimulate identification of novel biomarkers that can be used for analysis of the human brain, both for histological analysis of post‐mortem brain tissue and for possible generation of new positron‐emission tomography (PET) ligands in the long term, which may be useful to more precisely analyze PVS enlargement in patients with SVD and other neurological disorders.

## Unraveling specific links between the vasculature and the CNS in genetic mouse models

As discussed above, loss or impairment of cell types in the CNS affects the vasculature, and conversely, vascular problems lead to a dysfunctional CNS. While there are obvious large‐scale neuronal and white matter changes as a result of stroke, there are, however, also effects in the CNS resulting from more subtle modulations of the vasculature. Identifying molecular mechanisms operating in the CNS–vascular communication and which “relay” injury information from the neural to the vascular part, and vice versa, is important. Cell type‐specific modulation of genes in genetic mouse models is increasingly used to address these issues. This field is still at an early stage, but progress in genome‐editing techniques combined with improved opportunities for tissue‐specific expression of CRE recombinase and specific genes facilitates advancement in making more precise mouse models, and below we provide some examples of progress in the area of genetically modified mice.

The NVU is an important node for CBF control and constitutes a physical interface between cells from the CNS and the vasculature (Fig 2). As such, it provides a potentially interesting node for CNS–vascular communication, and it is an emerging notion that neurons and astrocytes exert important control functions over pericytes and endothelial cells in the BBB. ATP and adenosine from neurons act on adenosine receptors on the vascular cells, and arachidonic acid derivatives (including 20‐HETE, see below) from astrocytes also influence the vascular cells. Expression of human apolipoprotein E (APOE) in astrocytes in the mouse (driven from the GFAP promoter) resulted in an increase in CypA expression in pericytes and loss of BBB integrity 160. In line with this, pericyte loss is accelerated in humans carrying the *APOE4* allele 88. In a recent study, expression of tau in neurons of mice (the Tg4510 mouse model) resulted in an altered cortical microvasculature, with elevated numbers of small‐diameter blood vessels 161.

Conversely, experimental manipulation of gene functions in vascular cells in the mouse affects the CNS. Removal of *Glut1* from the mouse endothelium causes BBB breakdown, accompanied by a worsened neuropathology and decline in cognitive function 162. In line with this, genetic *GLUT1* defects in humans give rise to infantile seizures, microcephaly, and ataxia, underscoring the importance of a functional BBB (see ref. 163 for review). Interestingly, repletion of Glut1 (by viral transduction) in *Glut1*‐deficient mice averts some of the microvascular defects 164. Gene perturbations specifically affecting pericytes and VSMC may be useful tools to study the impact of a primary vascular injury on the CNS. Pericyte deficiency (using the *Pdgfrb* ^*F7*/*F7*^ mouse, see above) leads to white matter degeneration, changes in oligodendrocyte number, and dilation of PVS at a time window where pericytes but not VSMC are affected 165. Furthermore, loss of pericytes reduces clearance of Aβ in a mouse model overexpressing APP 87, and pericyte‐deficient mice show signs of neurodegeneration and cognitive decline 38. Expression of a CADASIL‐mutated form of the *Notch3* gene in VSMC in the mouse results in white matter lesions, similar to those observed in CADASIL patients 166. In conclusion, these observations support the notion that specific genetic perturbations in the CNS or vascular system can affect other tissues and lend support to the fact that precise mouse models are important tools to investigate CNS–vascular communication mechanisms.

## Toward therapy development

Development of therapeutic strategies that can restore a damaged vasculature and BBB is a prioritized goal not only for cerebrovascular diseases but will likely also become a component of future neurodegenerative disease treatment. BBB leakage may occur through disturbed transcytosis or breakage of the junctions between endothelial cells, leading to paracellular leakage. In the first category, a recent report suggests that reduced N‐cadherin expression in pericytes increases transendothelial transport 167. The pericyte represents a cell type of particular therapeutic interest, given its critical role in controlling BBB integrity via regulation of endothelial barrier function. Interestingly, imatinib (Gleevec) has been shown to improve BBB integrity caused by pericyte deficiency 20, and cilostazol/iloprost reduces pericyte detachment in an experimental stroke model in rat 168. Pericytes exhibit plasticity after experimental injury 169, supporting the notion that their function may be improved or restored in an injury situation. The study of BBB integrity in *in vitro* systems could clearly accelerate research and allow high‐throughput screens to be conducted (for review see ref. 170). Although not perfect mimics of the *in vivo* situation, organoid‐based *in vitro* BBB systems for endothelium–pericyte interactions have recently been developed 171, 172. Such three‐dimensional tissue‐mimicking systems may be better approximations of the *in vivo* situation than their two‐dimensional counterparts, and it will be interesting to learn whether these systems will accelerate the discovery of therapeutic agents and provide new insights into mechanisms affecting BBB integrity. A complementary, although less high‐throughput, model system for drug screening is zebrafish, where the combination of transparent embryogenesis and new fluorescent reporters to identify the various vascular cell types allows BBB function and other aspects of vascular biology to be monitored *in vivo* 173 (for review see ref. 174).

Therapies improving the outcome after ischemic stroke are also warranted but altered BBB permeability is a critical issue. Directly after acute ischemic stroke, tissue plasminogen activator (tPA) is used for thrombolysis, but beyond a few hours‐long time window of therapeutic opportunity, tPA treatment is associated with intracerebral hemorrhage, a consequence of induced BBB breakdown. Recently, it has been demonstrated that PDGF signaling (PDGF‐CC/PRGFRα) may become induced downstream of tPA as a consequence of tPA‐mediated cleavage and activation of latent PDGF‐CC, and that modulation of PDGF may improve post‐stroke BBB function 175. The mechanism of PRGFRα−induced BBB disruption is not understood, but intriguingly, the expression of Pdgfra is largely restricted to perivascular fibroblasts among the different cell types of the NVU 17. Along similar lines, the tyrosine kinase inhibitor imatinib, an efficient inhibitor of PDGFR signaling, was reported to reduce neurological disability after ischemic stroke 176. As discussed above, pericytes are important for the constriction of capillaries and reduced CBF observed in the post‐stroke situation 39, 40. 20‐HETE has been shown to constrict pericytes 40, and thus represents an interesting potential therapeutic target. As pericyte contractility is controlled by intracellular Ca^2+^ levels, modulation of the Ca^2+^ levels represents another interesting therapeutic possibility, although the overall efficacy of this approach still remains to be proven. An alternative approach is based on focused ultrasound, which may transiently open BBB to release toxic Aβ from AD patients 177.

While restoration of the BBB is the goal in disease situations characterized by an aberrant and leaky BBB, there are also situations where transiently enhanced BBB permeability could be advantageous. The barrier function of the BBB currently poses a problem for many therapeutic agents to effectively be transported from the blood to the brain, for example, in the treatment of brain tumors such as glioblastoma. Novel strategies to selectively enhance BBB permeability, possibly by transiently inhibiting pericyte function, can be considered. Approaches enhancing transcytosis have been attempted in the area of immunization‐based AD therapies, for example, by using transferrin receptor‐mediated transcytosis of anti‐Aβ antibodies using a bivalent antibody strategy 178, 179, and may be considered also to modulate pericyte function. To enhance paracellular transport by transiently disrupting the tight junction complexes is an alternative strategy, and in mice with cerebral edema, reduced swelling and cognitive improvement were observed upon siRNA knockdown of Cldn5 180. The beneficial effect of Cldn5 knockdown may seem paradoxical given that full knockout of the gene in mice is lethal 36; however, a complete breakdown of endothelial junctions is not observed in these mice 36. Cldn5 modulation may therefore be an interesting therapeutic avenue to resolve brain edema or allow transient passage of drugs. However, despite its fundamental importance for the BBB, the Cldn5 and tight junction dynamics at the BBB are still poorly understood, and the possibility of exploiting this molecule for therapeutic purposes requires further study.

## Concluding remarks

Neurodegenerative and cerebrovascular diseases have major medical, social, and economic consequences. Although progress is made in identifying pathomechanisms for these disease categories, it is an emerging notion that there are several links between neurodegenerative and cerebrovascular diseases, and to understand these links may provide new angles for therapy development and for a more complete understanding of the disease process. A central cell type at the interface between the CNS and vasculature is the pericyte, which is a universal component of the microvasculature, but nevertheless seems to play specific roles for the BBB. Investigation into the organ‐specific specialization of pericytes is still at its infancy, but data collected thus far point to the presence of specific functions for brain pericytes 17. Changes in pericyte function may have immediate consequences for CBF and BBB function, and therefore, to deconvolute how vascular dysfunction may cause, for example, white matter lesions will be important topics for future study. There are additional outstanding questions in this area, including whether there are qualitative differences in the microvasculature in different brain areas and whether pericyte–endothelial interactions differ, for example, between gray and white matter. While the available data do not indicate extensive brain pericyte heterogeneity at the transcriptomic level, more focused, brain region‐specific analyses need to be undertaken. With increasing knowledge about molecular mechanisms operating in pericytes, and in the contact between pericytes and other cell types, new possibilities to control pericyte function may be envisaged. There are situations where pericyte function would need to be restored, but also situations where a transiently induced pericyte dysfunction could be beneficial, for example, to increase access to the brain for drug delivery or to decrease brain edema. With recent insights into the transcriptional nature of pericytes and other vascular cell types in the brain, research in these areas may be further accelerated, and it will be interesting to witness the developments over the next few years.

**Box 1: In need of answers**

- What is the molecular nature of pericytes? Establishing the molecular nature of pericytes in different brain regions and across different organs will be important to understand the full spectrum of pericyte intra‐ and inter‐organ molecular heterogeneity. Progress in single‐cell transcriptomics combined with new strategies to sort pericytes will aid in this process.
- What is the function of pericytes in paravascular flow? How fluids (interstitial and cerebrospinal fluid; ISF and CSF, respectively) are transported to the brain is yet poorly understood, but evidence for a role of pericytes is emerging. Precise genetic perturbations in pericytes of the mouse combined with emerging techniques to record brain fluid transport may shed further light on this topic.
- How do neurodegenerative diseases affect pericytes? It is increasingly apparent that pericyte function is damaged by neurodegenerative diseases, but the molecular underpinnings of this dysregulation are not fully understood. Progress in the analysis of the vasculature in genetic mouse models for neurodegenerative disease and in post‐mortem material from patients suffering from neurodegenerative disease may provide new important insights.
- How do pericytes change the properties of the blood–brain barrier? To find ways for how to tune pericyte function in the blood–brain barrier to regulate permeability will be important. To enhance blood–brain barrier integrity would be advantageous, for example, after stroke and potentially also in AD, while a transiently increased transcytosis across the blood–brain barrier may facilitate uptake of drugs from the vasculature into the brain.
